# Peritoneal Dialysis in Diabetics: There Is Room for More

**DOI:** 10.4061/2011/914849

**Published:** 2011-10-16

**Authors:** P. Cotovio, A. Rocha, A. Rodrigues

**Affiliations:** ^1^Nephrology Department, Centro Hospitalar de Coimbra (CHC), Quinta dos Vales, 3041-801 S. Martinho do Bispo, Portugal; ^2^Nephrology Department, Centro Hospitalar do Porto (CHP), Largo Prof. Abel Salazar, 4099-001 Porto, Portugal

## Abstract

End stage renal disease diabetic patients suffer from worse clinical outcomes under dialysis-independently of modality. Peritoneal dialysis offers them the advantages of home therapy while sparing their frail vascular capital and preserving residual renal function. Other benefits and potential risks deserve discussion. Predialysis intervention with early nephrology referral, patient education, and multidisciplinary support are recommended. Skilled and updated peritoneal dialysis protocols must be prescribed to assure better survival. Optimized volume control, glucose-sparing peritoneal dialysis regimens, and elective use of icodextrin are key therapy strategies. Nutritional evaluation and support, preferential use of low-glucose degradation products solutions, and prescription of renin-angiotensin-aldosterone system acting drugs should also be part of the panel to improve diabetic care under peritoneal dialysis.

## 1. Diabetes Mellitus as a Leading Cause of End-Stage Renal Disease

End-stage renal disease (ESRD) can be considered a health epidemic involving considerable human and financial resources [1, 2]. The number of patients with ESRD is increasing in the world due to aging populations, longer life expectancy, increasing access to renal replacement therapies (RRT), and higher incidence of diabetes mellitus (DM) and hypertension. Nowadays, dialysis is the dominating therapy to prevent death from uremia, in large part because donor kidneys are in short supply, and thus, the survival of these patients is still a major concern [3]. According to the United States Renal Data System (USRDS), in 2008, the adjusted rate of prevalent ESRD cases rose 1.9%, to 1.699 per million population (pmp), with 547.982 patients under treatment. The prevalent dialysis population increased 3.6%, reaching 382.343 patients and has grown 34.7% since 2000 [4]. Among these amazing numbers, DM is present as the leading cause of ESRD in the USA and most other countries. After a dramatic increase in the incidence rate of ESRD due to diabetes, peaking in 2006 at 160 pmp, this rate fell 3.2% and 1.5% in the following two years, reaching 153 pmp in 2008, but still corresponding to 43% of all incident patients [4]. 

Although their survival is still much worse than that of nondiabetic counterparts, mainly because of the preexisting severely compromised cardiovascular conditions, between 1994–1998 and 1999–2003, the 5-year diabetic patients survival improved 15.3% in hemodialysis (HD) and 27.1% in peritoneal dialysis (PD), reaching 29% and 27%, respectively [4]. In Europe, diabetes as the cause of ESRD averaged 124 pmp. In the cohort 1999–2003, the unadjusted 1-, 2- and 5-year survival of patients on RRT was 80.8% (95% CI: 80.6–81.0), 69.1% (95% CI: 68.9–69.3), and 46.1% (95% CI: 45.9–46.3), respectively. Survival of incident diabetic patients either in HD and PD was the lowest and around 30% by 5 years [5].

## 2. Potential Benefits of Peritoneal Dialysis in Diabetics

Global benefits of home therapy [6, 7] and mainly slow sustained ultrafiltration (UF) conferred by PD [8] are particularly important in diabetic uremic patients. Even before the dialysis stage, most diabetic patients with ESRD have multiple cardiovascular and metabolic complications. Because of the rapid and intermittent removal of solutes and water and the extracorporeal circulation inherent to HD, it can frequently be associated with dialysis-induced hypotension, coronary ischemia, and arrhythmia [9], possibly leading to a worsening cardiovascular status in these patients [10]. A recurrent circulatory stress is postulated as a cause of important deleterious effects of standard schedules of HD [11]. On the contrary, PD avoids aggressive fluid shifts offering a better hemodynamic tolerance. It deserves to be mentioned that HD-induced myocardial stunning is identified as a new aspect of cardiovascular disease (CVD) in chronic kidney disease (CKD) [12, 13], while PD is not associated with such complications [14]. In addition, the lack of a need to create an arteriovenous fistula, which increases cardiac load, accelerating heart failure, may also be a potential benefit of PD in diabetic patients [10]. The preservation of the vascular network, usually frail in these patients, and sparing diabetic patients from the serious complications of vascular access thrombosis and infections are certainly underestimated but crucial arguments favoring PD in diabetics. 

PD also protects patients from the HD-induced recurrent regional ischemia that may lead to increased endotoxin translocation from the gut. Resultant endotoxemia is associated with systemic inflammation, markers of malnutrition, cardiac injury, and reduced survival. Circulating endotoxemia was most notably documented in those patients with the highest CVD, and a sharp increase was observed after initiation of HD [15]. 

Besides, residual renal function (RRF) loss threatens patient survival both in HD and PD [16]. Diabetes is also a risk factor for faster RRF decline [17]. However, PD might confer more RRF protection [18–22] in this group of risk patients. Moist et al. showed that the risk of RRF loss was 65% lower in PD patients than in HD [23]. Furthermore, they showed that the selection of HD as the dialysis modality and diabetic nephropathy were predictors of RRF loss. Thus, the approach of “PD first” appears to be a rational way to maximize the maintenance of RRF in diabetics, while simultaneously avoiding instrumenting a frail vascular capital and exposing patients to the risk of aggressive fluid shifts. On the other hand, RRF preservation and the consequenced higher elimination of advanced glycated proteins may overcome the risk of glucose degradation products (GDP) accumulation as a deleterious effect of PD in diabetics. 

Other potential advantages can still be mentioned. Fewer episodes of progressive diabetic retinopathy were observed in the PD patients, also fewer events of hemorrhagic retinopathy, and this is probably related to a more stable hemodynamic status and a lack of exposure to heparin. In a Japanese study that evaluated the progression of retinal lesions in diabetic patients either on HD or PD treatment, no patients in the PD group showed worsening of diabetic retinopathy during a 1-year observation period, compared to approximately 20% of HD patients [24]. 

Concerning insulin therapy, the advantages of intraperitoneal insulin administration include a higher physiological effect of insulin in patients with diabetic nephropathy during continuous ambulatory PD (CAPD) or automated PD (APD) treatment. Major fluctuations of blood glucose, hyperinsulinemia, and the formation of insulin antibodies can be minimized. In the final analysis, a better adjustment of blood glucose levels results [25]. However, there are pros and cons of various forms of insulin administration therefore, this might not be a sound argument for PD by itself. The reduction in insulin requirement is most pronounced compared with subcutaneous administration when insulin is instilled into the empty abdominal cavity, but if insulin is instilled with dialysis solution, there are losses of activity due to adsorption to the plastic surface of delivery systems [25]. Besides, bioavailability of intraperitoneal insulin might differ according to the solution bag used [26]. Importantly, hepatic subcapsular steatosis may be a complication associated with intraperitoneal insulin [27]. As part of other general PD benefits that also address diabetics, further aspects can be added. PD also allows good hemoglobin targets maintenance at lower erythropoietin doses, with both clinical and economic advantages [28]. Patients on PD also have a lower risk of contracting certain blood-borne diseases, like hepatitis C, which constitutes another advantage of this modality. In a report by Pereira and Levey, the prevalence of antiHCV antibodies in patients on dialysis was significantly lower in PD than in HD patients [29]. Additionally, PD is associated with lower rates of delayed graft function after transplantation [30, 31], possibly due to lower risk of hypotension and hypervolemia particularly relevant in diabetic patients prone to hemodynamic intolerance.  Table 1 summarizes these general and specific PD benefits in diabetic patients.

## 3. Controversy on Survival Data

Despite the substantially equivalent survival in diabetic patients either on HD or on PD [32] and the good reasons for initially offering PD to this group, only a small and decreasing proportion of diabetics receive PD [33, 34]. There is not any *a priori* first-choice dialytic treatment modality for these patients, and the decision to adopt HD or PD should be made on medical grounds and, above all, on the wishes of the individual patients. Whichever the modality, diabetics suffer from further clinical complications. A number of earlier studies documented varied results, some already beneficial to PD in diabetic patients in spite of addressing a remote PD era with less therapy resources, others showing possible lower benefit from PD regimens in older diabetics [35–40]. A long-term PD favorable study, which included more than 400 patients, showed that the best survival occurred in nondiabetic patients on PD, the survival rate of diabetic patients on PD was equal to that of nondiabetic patients on HD, and diabetic patients on HD had the worst survival rate [10]. Subgroup analysis in specific populations might, however, alert for lower benefits or even increased mortality risk in older diabetic patients in PD modality [41]. 

Vonesh et al. systematically reviewed six large-scale registry studies and three prospective cohort studies that compared mortality among ESRD patients receiving HD versus PD, conducted in the US, Canada, Denmark, and The Netherlands. Generally, PD was associated with equal or better survival among nondiabetic patients and younger diabetic patients in all four countries, while among older diabetic patients, results varied by country. Among older diabetics, the Canadian and Danish registries found no difference in survival between PD and HD, while in the US, HD was associated with better survival only in diabetics aged 45 and older [42]. A more recent Dutch study reported that the survival advantage for PD compared with HD patients decreases over time, with age and in the presence of diabetes as primary disease [43]. Among 139 diabetic PD patients studied during a mean followup of 28.2 ± 21.8 months, Fang et al. found 1-, 2-, 3-, and 5-year patient survival rates of 91%, 76%, 66%, and 47%, respectively [44]. These outcomes were better than those reported by USRDS for incident diabetic PD patients (85.7%, 67.9%, 52.5%, and 26.0%, respectively), and the data reported by CORR (86.4%, 53.6%, and 31.3% at 1-, 3-, and 5-year). Diabetic patients had a significantly poorer survival rate than did nondiabetics, both in the group younger than 65 and in those patients aged 65 or older [44]. Two recent studies also showed higher mortality and hospitalization rates in diabetic versus non diabetic PD patients. The presence of more morbidity factors at starting PD and a higher rate of previous cardiovascular events in diabetic patients may explain part of this risk [45, 46]. Added reasons for the reported worse outcome might be the variations in fluid homeostasis and corporal composition in diabetic patients, as fluid overload is the main cause of death in ESRD dialysis patients, and fluid control is potentially more difficult in PD diabetic patients [47]. Adjusted therapy is mandatory since results might differ according to treatment skills and policies. Besides, differences of some months of survival might be statistically significant but not clinically relevant. In fact population-averaged survival curves comparing adjusted PD and HD survival for US Medicare patients (1995–2000), showing that adjusted median life expectancy in HD is 35.1 months and in PD 33.8 months, are such an example [42]. 

More recent cohorts safely support PD prescription for diabetic patients, demonstrating similar long-term patient survival in both modalities and that DM *per se* should not be a barrier to PD [48]. Instead, the higher mortality rate in diabetic PD patients, in particular among female patients, was mainly attributable to concurrent morbidity such as CVD and protein-energy wasting, together with low RRF [49]. PD is more beneficial as the initial modality of dialysis for ESRD patients. Older patients with diabetes and patients without diabetes may switch modality to HD or undergo kidney transplantation in 1-2 years' time; long-term PD is viable in younger patients with diabetes [50]. 

## 4. Peritoneal Dialysis Risks in Diabetics

Glucose and insulin homeostasis are altered in CKD patients even in the early stages of renal disease. Metabolic syndrome is usually defined as a cluster of risk factors—obesity, high blood pressure, insulin resistance, and dyslipidemia—that are involved in development of CKD or are a consequence of it. It is argued that uremic patients treated with PD have a higher risk for deregulated metabolic response because of increased glucose absorption, with hyperglycemia prevalence greater than 50% comparing to 20% in HD patients [51]. It was also found that metabolic syndrome is a potent risk marker for adverse CV outcomes in nondiabetic patients on PD [52]. Notably, 60–80% of glucose-containing PD solution instilled into the peritoneal cavity is absorbed, corresponding to daily intake of 100–300 g glucose. This continuous glucose absorption modulated also by uremic toxicity and factors related to PD fluid bioincompatibility may indeed lead to aggravation of hyperglycemia, obesity and hyperlipidemia. Al these factors trigger the production of reactive oxygen species (ROS) and induce an inflammatory cascade that includes blocking insulin action and normal lipoprotein metabolism as recently revised [53]. Waist circumference is not a correct parameter to evaluate obesity due to the presence of the Tenckhoff catheter and potential residual peritoneal dialysate inside the abdominal cavity; however the use of body mass index (BMI) is also a biasing factor. Increased body mass due mainly to fat has a different prognostic meaning than body mass related to more muscle. PD patients with a high BMI associated with high muscular mass seem to have a survival advantage, compared with those with high BMI but low muscular mass that have an enhanced risk of cardiovascular death [54]. 

It is well established that PD patients frequently gain weight (fat mass), especially during the first year of PD therapy and particularly if they have diabetes or have a high BMI at initiation. However, several groups have not found any association between glucose absorption and weight gain. However the application of icodextrin solution may be a better option to alleviate excessive fat gain over time for patients on PD with studies revealing a significantly lower percentage of fat mass during the first 36 months (*P* < 0.05) [55]. Factors associated with the higher percentage of fat mass gain over time on PD were age, diabetes, gender (female) and nonicodextrin group (all, *P* < 0.01, generalized estimating equation). Wang and his/her group also found that genetic factors play an important role in the accumulation of fat mass in PD patients (uncoupling protein 2 exon 8 insertion/deletion polymorphism) [56]. Concerning to hyperglycemia diagnosis and glycated hemoglobin levels, important factors must be mentioned, because deregulation of sugar levels, associated with glucose-containing solutions, has important non linear implications for the patients. It has been documented that after 18 months of followup of 269 nondiabetic PD patients, HbA1c was the significant risk factor for all-cause mortality after relating variables were adjusted (HR: 4.114; 95% CI: 1.426–11.872; *P* = 0.009). Moreover, high HbA1c (HR: 3.892; 95% CI: 1.273–11.959; *P* = 0.026) and low HbA1c (HR: 1.179; 95% CI: 1.160–1.198; *P* = 0.039), with middle HbA1c group as the reference, also significantly predicted for mortality in these patients [57]. However, PD patients had lower HbA1c values than those without CKD with the same average glucose level, suggesting that HbA1c underestimates the glucose level in these patients. This underestimation might be secondary to the use of erythropoietin, meaning that a larger proportion of circulating erythrocytes have not been around long enough for sufficient glycosylation of hemoglobin. It was shown that a greater predictive value is achieved with the use of glycated albumin which measures glycemic control over the preceding 2 weeks and is not affected by serum albumin concentrations [58]. Furthermore, PD patients are never truly fasting because they continuously absorb glucose from the PD solution. So it is important to consider the limitations of both HbA1c and home glucose monitoring in PD patients before one makes therapeutic decisions. However, our goal is to achieve an HbA1c of <7% [59], taking into account that strict glycemic control based in such parameter might be hazardous in individual patients [60]. 

Blood glucose measurements in patients receiving icodextrin must be done with a glucose-specific method to avoid interference by maltose, a metabolite of icodextrin. 

Glucose dehydrogenase pyrroloquinoline quinone or glucose dye oxidoreductase-based methods must not be used. Results inconsistent with clinical suspicion of hypoglycemic coma should be retested with another testing system [61, 62]. Therapy must also be adjusted according to PD regimens aiming for glucose sparing prescriptions. Diabetic patients have a minimal increase in insulin requirement after initiation of PD *per se*, but the dosage of insulin increases markedly after exposure to hypertonic glucose solution [63]. A study of risk factors for high glucose use in PD patients showed that patients with DM, high BMI, and low RRF were more likely to require a high glucose load for PD therapy, especially during the first 3 years. After that, DM was the only significant factor associated with the need for higher glucose load [64]. One of the implications associated with hyperglycemia is the activation of the thirst mechanism with problems in managing fluid balance. Devolder et al. evaluated volume status with multifrequency bioimpedance (body composition monitoring, bioimpedance analysis (BIA)) in PD and HD patients revealing in multivariate models that diabetes and being under PD are associated with increased extracellular water (ECW) volume [65]. In turn, fluid overload implies use of more hypertonic bags negatively impacting glycemic control and peritoneal integrity, therefore creating a vicious circle. A baseline fast transport status can also be present in diabetics which will oblige careful therapy adjustment [66]. Peritoneal membrane exposed to higher content of GDP present in PD solutions is impaired by several mechanisms. First, GDP activates an inflammatory process promoting neoangiogenesis and consequently fast transport proprieties in the face of a progressive increase in peritoneal permeability. As a result of the inflammation, profibrotic factors are generated, such as transforming growth factor beta, leading to peritoneal fibrosis and accelerated loss of UF, ultimately leading to possible technique failure in diabetic patients on PD [10]. 

It is well known that diabetics have impaired antibacterial defenses and the risk of dying from an infection increases with worse glycemic control. It was demonstrated that GDP rich solutions accelerate leucocyte apoptosis and adversely affect the peritoneal defense [67]. However, neither peritonitis episodes nor other PD related infections have been observed to be more common in diabetic than in nondiabetic patients [47]. Several articles report, notwithstanding the risks presented, no difference in technique survival between diabetics and nondiabetic dialysis group of patients [49]. Clinicians should however be most concerned about nutritional and volume status of PD diabetic patients, which impact on patient survival. The etiology of malnutrition is multifactorial (acidosis, insulin resistance, inflammation, dialysate protein losses) and delayed gastric emptying associated with autonomic nervous system dysfunction is considered to be a significant factor. Dialysate volume in the peritoneal cavity was initially appointed as having a negative effect on gastric emptying. But in clinical practice its removal was not associated with any noticeable improvement. A study designed to estimate the direct influence of indwelling dialysate in the peritoneal cavity on gastric emptying, in patients treated with CAPD, determined by dynamic abdominal scintigraphy have shown no significant differences between those with and without indwelling dialysate. However, gastric emptying is markedly impaired in CAPD patients compared to healthy subjects indicating that other factors are responsible for the development of gastropathy in these patients, suggesting a gastric motility test with an empty peritoneal cavity [68]. A recent report, using an extracellular mass/body cell mass ratio (ECM/BCM ratio) as a marker of malnutrition, revealed that for every 10% increase of it, the relative risk of death was increased by about 35%, demonstrating that bioimpedance-derived enrollment ECM/BCM ratio was an independent predictor of long-term survival in PD patients [69]. Paniagua and his group studied the role of N-terminal fragment of B-type natriuretic peptide (NT-proBNP) as a predictor of mortality in ESRD patients. Showing that NT-proBNP levels and ECW/total body water (TBW) were correlated with several inflammation, malnutrition, and myocardial damage markers, they proved that NT-proBNP might be an added reliable predictor of death risk independently of the effect of dialysis modality [70]. 

Multifrequency BIA also enabled documenting that ECW adjusted for height was similar in diabetic and nondiabetic patients, but the ratio of ECW to TBW was greater for diabetics [71]. Additionally, a prospective study that evaluated heart failure in long-term PD patients concluded that diabetes and left ventricular mass and volume index were significant predictors of new-onset heart failure [72]. On the other hand the status of peritoneal fast transport, if inadequately managed, is also associated with worse outcomes and diabetes has been associated with a higher proportion of fast transport status. Icodextrin and APD can adequately support these patients. However there is concern about peritoneal protein leak during continuous PD procedure [73], because although correlated with volume overload and inflammation, it is largely an independent predictor of mortality [74]. Increased large-pore protein loss may reflect the severity of underlying CVD, portending a poor prognosis for these patients. Peritoneal protein clearance and not peritoneal membrane transport status predicts survival in contemporary PD patients [75]. Additionally higher daily peritoneal protein clearance when initiating PD was independently associated with peripheral arterial disease, a possible new marker of systemic endothelial dysfunction [76]. Last but not least, there are concerns about PD modality and transplantation outcomes. Analysis of the risk factors for development of posttransplant DM (PTDM) was performed with respect to pre-transplant dialysis modality. A total of 137 (6.8%) patients developed PTDM; 7% in the HD group and 6.5% in the PD (*P* = 0.85). In the multivariate analysis, age, BMI, rejection episodes and use of tacrolimus were identified as independent risk factors for its development. Adjusted analysis confirmed that pre-transplant dialysis modality does not have an impact on the subsequent development of PTDM [77]. Likewise, PD prior to simultaneous-pancreas-kidney transplantation is not associated with increased incidence of intra-abdominal infection compared to HD [78]. As described, PD may provide several advantages for diabetic patients, while risks should also be taken into account to optimize therapy (Table 1).

## 5. Strategies to Improve Outcomes

Throughout this paper we have emphasized the importance of preservation of RRF attending to the advantage in controlling fluid balance and solute clearance, protecting patient life. This task force should be pursuit both before and after dialysis induction. Early nephrology referral is an important measure for improved long-term clinical outcome in type II diabetics on maintenance PD [79]. Indeed attending an options class predialysis was associated with more frequent selection of home dialysis, fewer tunneled HD catheters and lower mortality risk during the first 90 days of dialysis therapy [80]. Considering the timing of dialysis, there is however no benefit from an early start. A retrospective analysis of patients, entering the USRDS database from January 1, 1995 to September 30, 2006 was carried out, sorting patients into groups by estimated glomerular filtration rate (eGFR) at dialysis initiation. In this total incident population (*n* = 896,546), 99,231 patients had an early dialysis start (eGFR >15 mL/min per 1.73 m2) and 113,510 had a late start (eGFR ≤ 5 mL/min per 1.73 m2). The first group had increased risk of mortality, while the late start was associated with reduced risk of mortality [81]. In another study planned early initiation of dialysis (eGFR was 10.0 to 14.0 mL/min) in patients with stage V CKD in comparison with later stage (eGFR was 5.0 to 7.0 mL/min) was not associated with an improvement in survival or clinical outcomes. During a median followup period of 3.59 years, 152 of 404 patients in the early-start group (37.6%) and 155 of 424 in the late-start group (36.6%) died (HZ with early initiation, 1.04; 95% CI, 0.83 to 1.30; *P* = 0.75) [82]. An individualized strategy leveling indication and risk for dialysis induction in diabetics is mandatory. Dedicated and multidisciplinary care is than essential to offer the best treatment and the adequate control of cardiovascular risk factors: diet, exercise and weight control. Adequate patient education, and support with dietitian, podologist, endocrinologist and frequent monitorization are very important. One of the limitations is severe visual or functional impairment, necessitating the involvement of a relative. Experience has demonstrated that even blind patients can perform the technique properly, although it might oblige individualized and more prolonged training. Elective assisted PD might be considered in patients with less autodialysis capacity. There are multifactorial interventions that may additionally improve the survival of diabetic PD patients. 

New PD solutions with low GDP are promising in reducing the risk of CVD in these patients, by preserving RRF, optimizing volume control, and possibly reducing local and systemic inflammation. 

A study comparing the use of balance, a neutral pH, low GDP solution to conventional one, during 12 months, revealed a lower degree of systemic inflammation. The Balance group had a superior profile of PD effluent markers of mesothelial cell mass marker and inflammation [83]. 

As a glucose sparing policy, the use of 1 bag of icodextrin or amino-acid (AA) solution daily may reduce the glucose load by 15–30%. In addition, icodextrin use was significantly associated with a reduced risk of death (HR, 0.40; 95% CI, 0.28 to 0.58; *P* < 0.001) in a recent retrospective investigation. AA-containing dialysate has been used to compensate for a low dietary protein intake but clinical benefits have not been consistently demonstrated because these solutions can induce an anabolic response in malnourished patients on CAPD only if enough calories are ingested simultaneously. Dialysis solutions containing a mixture of AAs and glucose in appropriate proportions can serve as a source of both proteins and calories. Although the metabolism of intraperitoneal AAs causes the generation of hydrogen ions and urea, acid-base homeostasis can be preserved using dialysis solutions with a buffer content of 40 mmol/L [84]. Additionally, calcium exposure through PD solution plays a role in the progression of arterial stiffness, which may be related to increased vascular calcification [85], therefore individualized low calcium solutions should be prescribed. Most importantly icodextrine has a role in diabetic patients' PD treatment: as Paniagua et al. state, in a study of 12 months, the use of icodextrin allows in the early phase (6 months), reduction in ambulatory blood pressure (ABP) and left ventricular end diastolic diameter. These changes correlated with changes in body fluids. In the late phase (12 months), a trend towards baseline values in ABP was seen. It was argued that changes in inferior vena cava diameter and in low-frequency R-R variability spectral analysis suggest that icodextrin increases circulating blood volume and sympathetic tone, probably by accumulation of icodextrin metabolites in the bloodstream and improvement in diabetic neuropathy as a result of lower peritoneal glucose absorption [86]. 

The benefits of icodextrin use in diabetic patients were also supported by the same investigators in a prospective, randomized controlled trial, comparing it with conventional glucose solutions. These authors showed that icodextrin significantly reduces ECW volume, thereby leading to a significant reduction in both systolic and diastolic blood pressure. Moreover, they demonstrated that icodextrin, reduces blood glucose concentration, a finding that was accompanied by a concomitant reduction in insulin dosage. Furthermore, a particularly finding was that icodextrin was associated with a delay in the decline of eGFR and urine volume over a 6-month observation period [87]. So icodextrin, besides its elective indication in fast transporters, gathers a group of potential benefits in diabetic PD patients: increase in UF volume with better blood pressure control; increase in solute clearance; better glycemic control with fewer requirements for insulin; and better preservation of RRF. 

A bimodal solution based on the mixing of glucose (2.6%) and icodextrin (6.8%), during a 4-month prospective intervention period, showed that net UF and peritoneal sodium removal during the long dwell was about 2-fold higher than baseline. Therefore a combined crystalloid and colloid PD solution might be useful as a glucose-sparing strategy for volume control in high-transport APD diabetic patients [88]. Low-GDP solutions also are advocated, if financial constraints are not superimposed: in vitro and ex vivo studies clearly support better biocompatibility of these solutions. Notwithstanding, in the clinical field it was mainly RRF the parameter that showed to be protected by using these solutions. In a multicenter approach, 80 patients were randomized to treatment with a PD fluid containing low levels of GDP or standard PD fluid for 18 months. Data revealed a significant difference in monthly RRF change and twenty-four-hour urine volume decline, demonstrating a significant benefit concerning preservation of RRF and urine volume of using a PD fluid with low GDP levels [89]. The previously mentioned inflammatory cascade, activated by GDP is mediated by renin-angiotensin-aldosterone system (RAAS), constitutively expressed in peritoneal mesothelial cells, and ROS. So it is postulated that use of angiotensin converting-enzyme inhibitor (ACEI) or angiotensin II receptor blocker (ARB) helps, not only to preserve RRF in ESRD patients, but also to maintain peritoneal membrane integrity longer in PD patients. Thus, these classes of drugs should be the first choice for antihypertensive therapy [59]. Antioxidants, namely N-acetylcystein, might also support preservation of peritoneal membrane function by the same mechanism [50] but need further evidence.  Table 2 resumes strategies that potentially will improve clinical outcomes in PD diabetic patients. 

## 6. Our Clinical Experience of PD in Diabetic CKD

In our centre, by carrying out a prospective registry based study of 432 adult incident patients admitted during 25 years in a PD university program (11640 months at risk), we compared clinical outcomes of PD treatment on diabetic versus nondiabetic counterparts. At baseline analyzed groups were identical concerning mean age, proportion of older patients, gender, previous RRT and reason for PD; diabetic had lower RRT vintage (33(15–99) versus 75(23–125) months; *P* = 0.05). Patient survival was significantly lower in diabetic, when compared to nondiabetic: 89%, 77%, 67%, 52% versus 93%, 86%, 79%, 71%, at 1, 2, 3, and 4 years, respectively (*P* < 0.0001). However, technique survival was similar between diabetic and nondiabetic: 84%, 74%, 66%, 51% versus 87%, 79%, 66%, 57%, at 1, 2, 3, and 4 years, respectively (*P* = NS). Our results compare favourably with international reports: In EDTA registry report 2008 [5], diabetic patient survival was 73% by 2 years; in the French PD registry diabetic patients lower three-year patient and technique survival was reported as 59% and 34%, respectively for patients aged 50–60 years [90]. 

On multivariate analysis including in the model diabetes, older status, PD after HD and PD after transplant, DM was an independent predictor for patient mortality (HZ 2.3; CI 1.5–3.7) but not for technique failure (HZ 1.3; CI 0.9–1.9). A lower proportion of diabetics received a renal graft during the followup (19% versus 32%; *P* = 0.016). Among the reasons for transfer to HD, UF failure/underdialysis was similar between groups (26% versus 22%, *P* = NS), a higher proportion of dropout was observed in diabetic due to loss of autonomy for the technique (23% versus 5%, *P* = 0.004). The global peritonitis rate was similar between the diabetic and their counterparts: 0.53 versus 0.61 ep./pt.y (*P* = NS). The global hospitalization rate was significantly higher in diabetics than in nondiabetics: 1.39 versus 0.84 ep./pt.y (*P* = 0.004). In our study, PD proved to be an effective long term RRT option in diabetics, without a higher rate of technique failure, UF failure or peritonitis. DM was however associated with higher mortality, higher autonomy loss, and higher hospitalization rate, enhancing the need for investigation and control of comorbidities with potential negative impact on these parameters.

## 7. Conclusion

Diabetes is among nephrological causes of ESRD that are associated with the worst diagnosis. Independently of the renal replacement therapy patient survival is limited and exposed to higher rate of complications and hospitalizations. PD however, offers a cluster of advantages in diabetic patients: besides amenable better life-style the modality avoids vascular access complications in patients with frail vascular capital, protects RRF, and allows higher hemodynamic stability with less myocardial stress and stunning, with slower progressive retinopathy. The disadvantages can be overcome by adequate care, glucose-sparing PD regimens, optimization of volume control and other protective measures like RAAS acting drugs and antioxidants to prolong the best quality of care while on PD. P. Cotovio and A. Rocha contributed equally to this paper.

## Figures and Tables

**Table 1:** Potential benefits and risks of PD in the treatment of diabetic patients.

General PD benefits	Specific PD benefits in diabetics	PD risks in diabetics
(1) Home-based continuous therapy	(1) Sustained daily ultrafiltration	(1) Fluid overload
(2) Advantages in lifestyle	(2) Better preservation of residual renal function	(2) Aggravated dysregulated metabolic response to glucose
(3) Avoids vascular access related infections	(3) Vascular capital preservation	(3) Hyperinsulinemia
(4) Avoids recurrent circulatory stress	(4) Avoids peripheral and coronary steal syndromes	(4) Central obesity
(5) Avoids myocardial stunning	(5) Fewer episodes of hypotension	(5) Dyslipidemia
(6) Fewer episodes of blood-borne disease	(6) Better blood pressure control	(6) Peritoneal albumin losses
(7) More liberal diet (in spite of fluid and Na restriction)	(7) No need for systemic anticoagulation	(7) Peritoneal infection
(8) Control of anemia with lower doses of erythropoietin	(8) Fewer episodes of progressive retinopathy	(8) Membrane fast transport status
(9) Lack of pain from needle puncture	(9) Feasibility of elective intraperitoneal insulin	
(10) Lower rate of delayed renal graft function		

**Table 2:** Strategies to improve clinical outcomes in PD diabetic patients.

Strategies	Practice
(1) Opportune nephrology referral	More than 3 months before dialysis initiation, ideally when GFR ≤ 30 mL/min
(2) Residual renal function protection	Avoidance of dye studies, nonsteroidal antiinflammatory drugs (including cyclooxygenase-2 inhibitors), aminoglycosides, and extracellular fluid depletion
(3) Control of cardiovascular risk factors	Diet counseling and promotion of physical activity to avoid obesity; pharmacologic therapy for hypertension atherogenic dyslipidemia, dysglycemia and prothrombotic state (ACE inhibitors, AII receptor antagonists, B blockers, statins, and aspirin)
(4) Patient education and multidisciplinary support	Group discussion and individual consultation (booklets, video, and interview) promotion of hometherapy and transplantation (both renal and renopancreatic) glycemic control optimization foot care and peripheral vascular evaluation ophthalmologist followup
PD specific strategies	
(5) Skilled volume evaluation and control	Panel of clinical evaluation (blood pressure, weight, and edemas), biomarker (pro BNP) and multifrequency BIA (longitudinal trends of body composition) high-dose furosemide fluid, and sodium restriction elective use of icodextrine and APD
(6) Preferential use of low GDP solutions, glucose sparing regimens, and individualized low calcium solutions	Avoidance of hypertonic bags use Bi/tri compartment bag solutions (low GDP) individualized low Ca solutions prescription “PEN” regimen: physioneal; extraneal; dianeal; “NEPP” regimen: 1 amino acid exchange, 1 icodextrin exchange, and 2 glucose bicarbonate/lactate exchanges as options
(7) Nutritional evaluation and support	Assessed by a panel: subjective global assessment (SGA), protein equivalent of nitrogen appearance (nPNA), serum albumin and lipid profile, multifrequency BIA diet counseling by nutritionist
	Enteric supplements (protifar as protein supplement) peritoneal supplement (nutrineal once day)
(8) Preferential use of RAAS acting drugs	ACEI and ARB as first antihypertensive drugs possible protective effects in peritoneal membrane status
(9) Optimize technique survival and opportune transfer to HD	International recommendations on peritoneal access management and prophylactic measures individualized training and retraining peritonitis rate systematic control and quality assessment individualized APD prescription depression assessment and specific management routine annual peritoneal membrane evaluation
